# Anterior Cruciate Ligament Reconstruction With Fresh Frozen Soft Tissue Allograft: A Retrospective Case Series

**DOI:** 10.7759/cureus.94884

**Published:** 2025-10-18

**Authors:** Gipson Samuel, JVS Vidyasagar, Darshan Bachani, Harinath Reddy, Prabhu Muthiahpandian, Pasupathy Palaniappan, Shylvia Christy

**Affiliations:** 1 Orthopaedics, Jawaharlal Institute of Postgraduate Medical Education and Research, Puducherry, IND; 2 Orthopaedics and Sports Medicine, Gleneagles AWARE Hospital, Hyderabad, IND; 3 Orthopaedics, Marengo CIMS Hospital, Ahmedabad, IND; 4 Orthopaedics, Santhiram Medical College, Nandyal, IND; 5 Obstetrics and Gynaecology, Jawaharlal Institute of Postgraduate Medical Education and Research, Puducherry, IND

**Keywords:** anterior cruciate ligament reconstruction (aclr), fresh frozen allografts, graft failure, graft rejection, india, one leg hop test, one-leg hop test

## Abstract

Objectives

The purpose of this study was to assess the outcomes of fresh frozen soft tissue allografts in anterior cruciate ligament reconstruction (ACLR) in India.

Methods

This retrospective case series involves patients who underwent ACLR with allografts between February 2015 and October 2015 at Aware Global Hospitals, Hyderabad, and were contacted in October 2016 for follow-up. At follow-up, these patients completed questionnaires and underwent clinical examination. A one-tailed paired t-test was used to assess the non-inferiority of the operated limb compared with the contralateral limb in the one-leg hop distance.

Results

Of the 14 patients, four (28.6%) developed fever and joint swelling in the immediate postoperative period. At repeat arthroscopy, two (50%) of these four patients had intact grafts, while the other two (50%) had necrosed grafts. Both patients with graft necrosis required revision ACLR. Excluding these two patients (14.3%), all the others were symptomatically, functionally, and clinically near normal at the last follow-up visit. There were no cases of re-rupture or disease transmission. The mean difference in one-leg hop distance between the operated and non-operated limbs was −1.92 inches (SD 2.47). The upper bound of the 90% one-sided confidence interval (−0.95 inches) was well below the non-inferiority margin of 4.3 inches (p<0.000001), demonstrating statistical non-inferiority.

Conclusion

Fresh frozen soft tissue allograft use produced satisfactory outcomes in our patients. In the Indian population, where most anterior cruciate ligament (ACL) injuries occur in non-athletic individuals, fresh frozen soft tissue allografts can be considered a standard graft material for ACLR.

## Introduction

Fresh frozen soft tissue allograft is one of several options for grafts in anterior cruciate ligament reconstruction (ACLR) [1]. The use of allografts in ACLR offers several advantages, including the absence of donor site morbidity, smaller incisions, shorter surgical duration, reduced tourniquet time, and earlier recovery of muscle strength [2]. However, disadvantages include the risk of disease transmission, immune reactions, longer incorporation time, and prolonged graft remodelling [3]. Some studies also report that allografts may not completely remodel and have structural properties inferior to autografts [4].

In developed countries, approximately 50% of ACLRs use allografts [5]. Evidence suggests that allografts yield poorer results in athletic populations, whereas in non-athletic populations, outcomes are comparable to autografts, with the added benefit of shorter recovery times [6]. In India, most ACL injuries occur in non-athletic individuals, often as a result of road traffic accidents (RTAs), making fresh frozen soft tissue allografts a viable graft option. However, the availability of allografts is limited due to the small number of tissue banks capable of procuring, processing, sterilizing, and preserving these grafts. Demonstrating good clinical outcomes with allografts may help raise awareness of the need to establish more tissue banks in the country.

Allografts have been used for many years. Earlier, sterilization was carried out using ethylene oxide, irradiation, Bio-Cleanse (RTI Surgical, Florida, USA), or other chemical methods [7]. These prior techniques of procurement, processing, sterilization, and preservation produced inferior results. In contrast, current techniques have shown comparable outcomes to autografts, with fewer infections and immune reactions [8].

The purpose of this study is to evaluate the outcomes of fresh frozen soft tissue allografts in ACLR. In addition to evaluating functional and patient-reported outcomes, a secondary objective was to describe postoperative complications following ACLR with fresh frozen soft tissue allografts.

## Materials and methods

Study design & population

This retrospective case series, with level 4 evidence [9], included all patients who underwent anterior cruciate ligament reconstruction (ACLR) using fresh frozen soft tissue allografts between February and October 2015 at Gleneagles AWARE Hospital, Hyderabad, India. Patients with multi-ligamentous knee injuries were excluded from the study. At follow-up, these patients were assessed for pain using the Visual Analog Scale (VAS), functional status and satisfaction via questionnaires [10], range of motion (ROM) using a goniometer, muscle power using the Medical Research Council (MRC) grading system, joint translation in millimetres (mm) using stability tests [11], and one-leg hop performance [12]. The authors used a custom-developed proforma in this study, incorporating concepts from established knee outcome measures [12-15]. Although formal validation was not performed, the tool underwent internal pilot testing on five patients (not included in the study cohort) to ensure clarity and feasibility. The proforma, along with the questionnaire, is original and created by the authors.

In the one-leg hop test, the patient stands on the affected leg behind a starting line and hops as far as possible, landing on the same foot and maintaining balance for three seconds. The distance from the starting line to the heel at landing is recorded. The test is repeated three times, and the average distance is calculated. The same procedure is performed for the opposite leg to determine the limb symmetry index (LSI). Measurements were recorded in a Microsoft Excel workbook (Microsoft Corp., Redmond, WA, US).

Methodology and statistical analysis

A convenience sampling method (non-probability sampling) was used. The study was designed as a paired, one-tailed non-inferiority analysis comparing the one-leg hop distance between the operated and non-operated limbs after allograft ACLR. The primary objective was to demonstrate that the operated limb was not clinically inferior to the non-operated limb by more than 10% in one-leg hop distance at a minimum follow-up of one year.

The non-inferiority margin was set at 10% of the average expected one-leg hop distance (approximately 43 inches). The 10% margin was chosen based on prior work indicating that a difference of approximately 10% in one-leg hop performance is considered clinically meaningful following ACLR [12]. The mean side-to-side difference was assumed to be 1 inch, with a standard deviation (SD) of paired differences estimated at 5 inches [12]. Using the sample size formula for a non-inferiority test with paired data (80% power, one-tailed alpha of 0.05), the calculated sample size was 14.

The null hypothesis (H₀) stated that the operated limb was inferior by ≥10%, while the alternative hypothesis (H₁) stated that it was non-inferior by more than 10%. Data were analyzed using a one-tailed paired t-test for non-inferiority. A 90% one-sided confidence interval (CI) was constructed for the mean difference, with non-inferiority concluded if the upper bound was less than the pre-specified margin. Statistical analysis was performed using IBM SPSS Statistics for Windows, Version 26 (Released 2019; IBM Corp., Armonk, New York, United States).

Procedure

Fresh frozen soft tissue allografts were obtained from a single government-authorized tissue bank at M.S. Ramaiah Medical College, Bangalore, India. Tendons were procured within 12 hours of death, extended to 48 hours if the body had been preserved in ice. Donors were confirmed to be free from transmissible diseases.

Tendons were harvested using standard techniques, cleared of muscle, washed with saline, and immersed in gentamycin solution for 10 minutes. The samples were then sent for histopathological examination and microbiological analysis to confirm the absence of pathogens. Processed tendons were preserved at −80°C in a deep freezer until use [16], following tissue bank protocols adapted from France.

**Figure 1:**
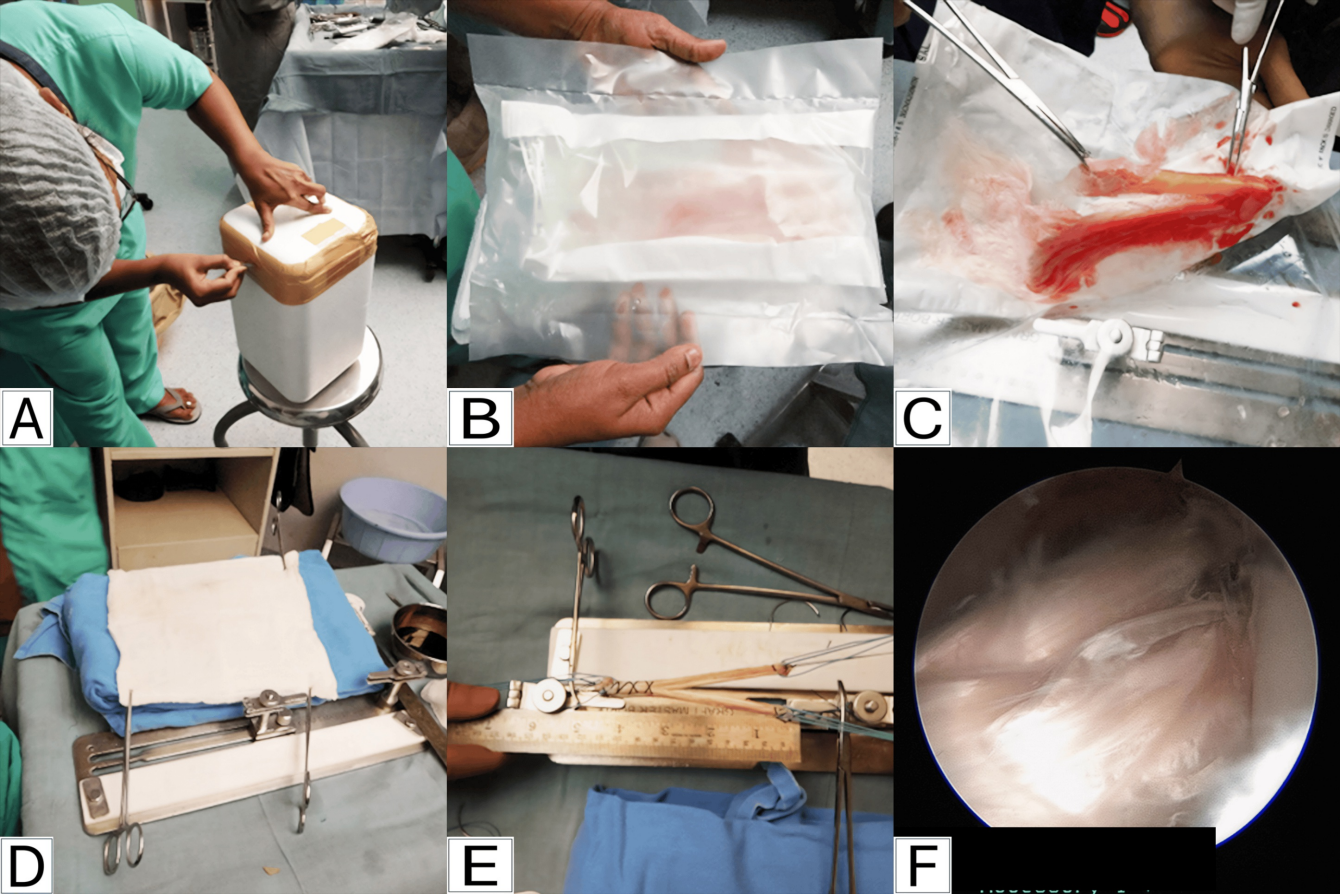
Stepwise preparation and use of tendoachilles allograft for double-bundle anterior cruciate ligament reconstruction (ACLR) A: Allograft received at -40°C; B: Allograft inside a triple sterile packing; C: Tendoachilles allograft removed from sterile packaging for thawing and preparation; D: Gradual thawing of allograft at room temperature; E: Tendoachilles allograft after preparation into a Y-shaped graft for double-bundle ACLR; F: Arthroscopic image showing the tendoachilles allograft Image credit: These are original intraoperative photographs taken by the authors during the surgical procedure.

All patients gave consent for ACLR with fresh frozen allografts and were operated on by a single senior surgeon. Preoperative and perioperative details were retrieved from the hospital information system. Intraoperatively, the allograft was received in a −40 °C storage container (Figure 1A), checked, and opened under aseptic conditions.

The triple sterile pack was removed (Figures 1B, 1C), and the graft was thawed at room temperature for about one hour (Figure 1D). After preparation (Figure 1E), it was passed through the tibial tunnel and fixed to the femoral tunnel under arthroscopic guidance (Figure 1F).

Rehabilitation began immediately after surgery and followed the standard protocol used for autograft ACLR. All patients were supervised by a single physiotherapist specialized in sports medicine. Early rehabilitation focused on pain control with ice packs and crepe bandage, patellar mobilization, quadriceps activation, and gradual range-of-motion exercises including assisted extension and flexion. By four to six weeks, patients progressed to full weight-bearing, sitting slides, cycling, and prone hamstring curls. From six weeks onwards, exercises included single-leg standing, swimming, and strengthening of hamstrings and adductors. At eight to 12 weeks, functional strengthening progressed to wall squats, straight-leg raises, extension holds, and extension curls. Once 65% limb strength was regained, advanced activities such as jump rope, jogging, and lunges were introduced.

There were no conflicts of interest. The patient proforma is provided in the Appendix.

## Results

Table 1 summarises the demographic variables, injury details, and intraoperative allograft details.

**Table 1:** Demographic, injury and surgery details (n=14) SD: standard deviation; HA: hydroxyapatite; TCP: tricalcium phosphate; RTA: Road traffic accident. *Tibialis anterior, tibialis posterior, Extensor Hallucis Longus (EHL) and Extensor Digitorum Longus (EDL)

Parameter	Frequency (n)	Percentage (%)
Age (years)		
Mean ± SD	30.9 ± 7.1	-
Range	19 to 41	-
Sex		
Male	14	100.0
Female	0	0.0
Chief complaints		
Pain and instability	14	100.0
Locking	1	7.1
Mode of injury		
RTA	8	57.1
Sports	4	28.6
Slip and Fall	2	14.3
Side affected		
Right	8	57.1
Left	6	42.9
Associated meniscus injury		
Lateral meniscus	7	50.0
Medial meniscus	4	28.6
Allograft used		
Peroneus Longus	3	21.4
Peroneus Brevis	3	21.4
Tendoachilles	2	14.3
Semitendinosus	2	14.3
Others*	4	28.6
Graft preparation technique		
Triple weave	7	50.0
Three tunnel double bundle	4	28.6
Others	3	21.4
Implant used for graft fixation		
Titanium screws	8	57.1
HA coated TCP	6	42.9

Patients presented with pain and instability of the affected knee at a mean duration of three months from the time of injury (range: 15 days to eight months). Among the four patients injured during sports, one (25%) was a 19-year-old amateur football player, while the other three (75%) sustained injuries during recreational activities.

Two patients (14.3%) developed anterior cruciate ligament (ACL) injuries following a fall; both had undergone ACLR for complete ACL tears 10 and six years earlier, respectively. Of the 14 patients, four (28.6%) had isolated ACL injuries, five (35.7%) had associated lateral meniscus injuries, three (21.4%) had associated medial meniscus injuries, one (7.1%) had both lateral and medial meniscus injuries, and one (7.1%) had a partial ACL injury.

For ACLR, tendoachilles grafts were used with the three-tunnel double-bundle technique [12]. Semitendinosus (ST), peroneus longus (PL), peroneus brevis (PB), tibialis anterior, and tibialis posterior tendons were used as triple weave grafts. The extensor hallucis longus (EHL) was used in one patient to augment a partially torn ACL. Aperture fixation was used in all cases.

Postoperative complications

**Figure 2:**
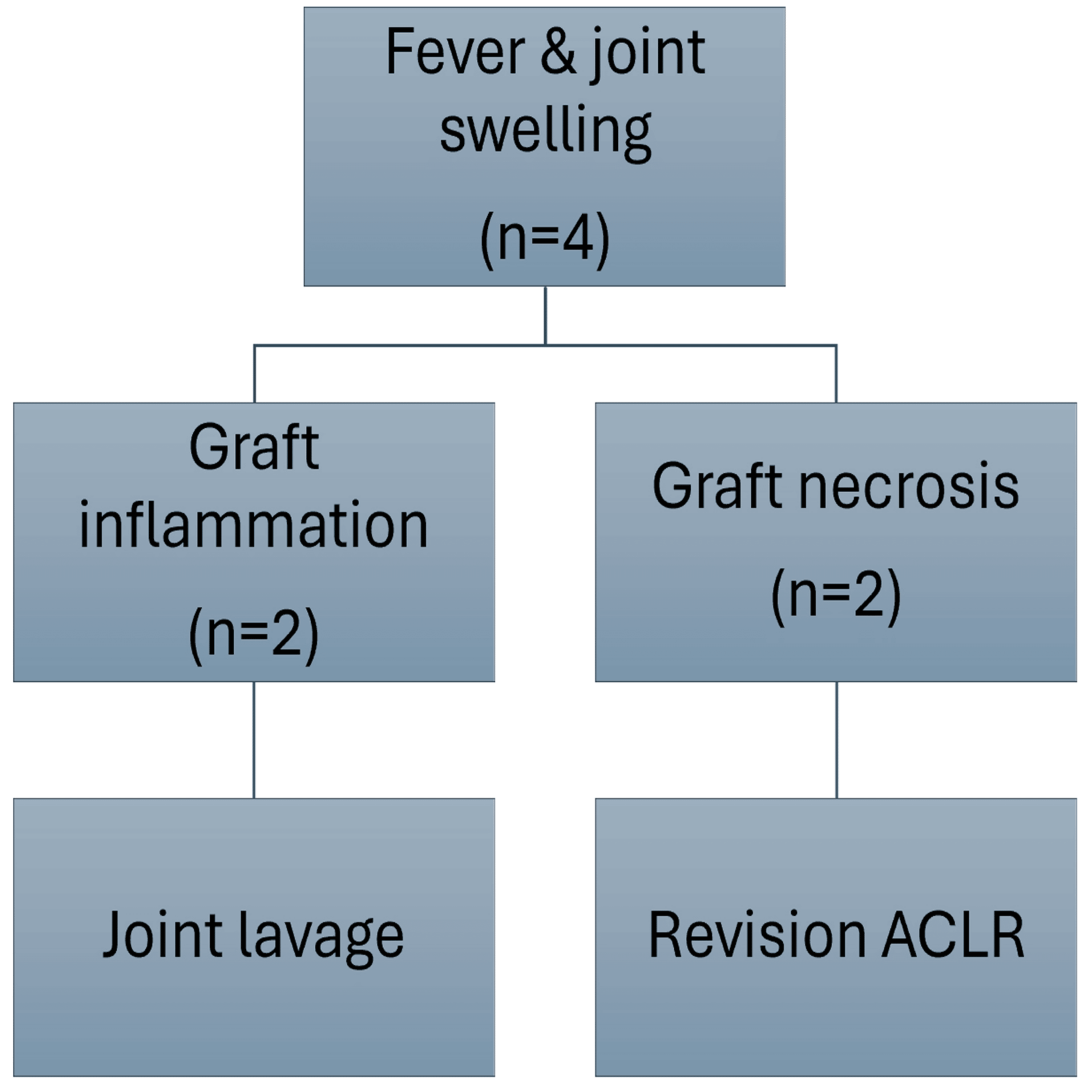
Patients with fever and joint swelling ACLR: Anterior Cruciate Ligament Reconstruction

Four patients (28.6%) developed fever and joint swelling in the immediate postoperative period. Two patients (50% of those with fever/swelling), a 41-year-old male with a left ACL injury, medial meniscus flap tear, and lateral meniscus posterior horn avulsion following a twisting sports injury two months prior, and a 30-year-old male with a right ACL injury and crushed lateral meniscus injury following an RTA four months prior, were managed with arthroscopic joint lavage. At arthroscopy, their grafts (Extensor Digitorum Longus (EDL) and PB, respectively) were intact, cultures were sterile, and biopsies showed synovitis. The other two patients (50% of those with fever/swelling), a 28-year-old male with a right ACL injury and lateral meniscus tear following an RTA five months prior, and a 37-year-old male with a left ACL injury and lateral meniscus tear following a twisting injury 1.5 months prior, had necrosed grafts (ST in both cases) on repeat arthroscopy. Cultures were sterile, and biopsies confirmed necrosis. Both underwent debridement followed by revision ACLR with contralateral ST autografts three months later. Figure 2 summarizes the clinical course of the patients with postoperative fever and joint swelling.

Follow-up outcomes

By October 2016, 10 patients (71%) had completed 18 months of follow-up, while four patients (28.6%) had completed 12 months, with a median follow-up duration of 18 months (IQR: 4.75). There was no loss to follow-up. At the final visit, no patient reported instability, locking, swelling, or weakness. All patients could walk, run, climb stairs, use public transport, ride two-wheelers, sit cross-legged, squat, and participate in recreational sports. Clinical examination showed normal ROM, normal quadriceps and hamstring strength, Lachman and anterior drawer tests comparable to the contralateral knee (<5 mm difference), negative pivot shift, and ability to perform the one-leg hop test. Table 2 summarizes the follow-up outcomes.

**Table 2:** Follow-up outcomes (n=12) IQR: Interquartile range

Follow-up outcome	Frequency	Percentage
Postop duration in months (median ± IQR)	18 ± 4.75	-
Occasional pain requiring rest	3	25.0
Difficulty in jogging up to 1 km	1	8.3
Difficulty in climbing stairs	0	0.0
Difficulty in sitting crossed leg	1	8.3
Difficulty in squatting	0	0.0
Participation in recreational sports	4	33.3
Satisfaction (n=14)		
Excellent	2	14.3
Good	8	57.1
Fair	2	14.3
Poor	2	14.3
Quadriceps weakness	0	0.0
Anterior drawer test (translation >5 mm)	0	0.0

Functional performance

Table 3 summarizes the one-leg hop test results. Analysis excluded the two patients with graft necrosis.

**Table 3:** One-leg hop test results (n = 12) ^1^Mean ± SD values are calculated for each limb from the average hop distances; ^2^Mean difference and its SD are calculated from the paired differences for each patient; ^3^p-value derived from a paired one-tailed t-test for non-inferiority with a margin of 4.3 inches.

Metric	Operated leg¹	Non-operated leg¹	Mean difference² (Operated − Non-operated)	p-value³ (one-tailed)
Mean distance (inches)	29.67	31.58	−1.92	<0.000001
SD (inches)	10.96	10.26	2.47²	—
Limb Symmetry Index (%)	92.96 ± 8.87	—	—	—

The mean difference between the operated and non-operated limbs was −1.92 inches (SD 2.47). A one-tailed paired t-test for non-inferiority yielded t=−8.73, p<0.000001. The upper bound of the 90% one-sided CI was −0.95 inches, below the non-inferiority margin of 4.3 inches, confirming statistical non-inferiority.

## Discussion

Two patients (14.3%) in this series were dissatisfied with their outcomes; both had culture-negative graft necrosis and required revision ACLR. Two others who developed postoperative culture-negative synovitis felt they might have done better with autografts. Excluding the two cases of graft necrosis, all fresh frozen soft tissue allografts were symptomatically, functionally, and clinically near normal. At the final follow-up, there were no graft ruptures or cases of disease transmission. The results from the 12 patients without graft necrosis are comparable to those reported in similar studies.

Mascarenhas et al., in a systematic review of overlapping meta-analyses comparing autografts and allografts for ACLR, concluded that current evidence shows no difference in rupture rates or clinical outcomes between the two. However, lower-quality meta-analyses suggest that autografts may result in lower recurrence rates, better hop test performance, and superior objective knee stability [17]. Song et al. found that hamstring autografts and soft tissue allografts have similar functional and radiological outcomes, but hamstring autografts showed fewer complications and better arthroscopic findings [18]. Zeng et al. reported that autografts offer advantages over irradiated allografts in function and stability, although no significant difference was found between autografts and non-irradiated allografts [19]. According to Lamblin et al., outcomes after autograft ACLR are comparable to those with non-chemically processed, non-irradiated allografts [20]. Similar findings were reported by Kan et al. [21]. Tejwani et al., in a study of primary allograft ACLR, concluded that irradiation above 1.8 Mrad, Bio-Cleanse processing, younger patient age, male sex, and bone-patellar tendon-bone (BPTB) allografts are associated with a higher risk of failure and revision surgery [22]. Guo et al. described culture-negative synovitis following ACLR with fresh frozen allografts, possibly due to immune rejection [23].

In our series, allograft use was well accepted by both donors and recipients in the Indian population when proper education was provided. Our study is limited by its small sample size (n=14), the use of convenience sampling, and the absence of a control group. The follow-up period of 12-18 months, while adequate for early outcome assessment, is insufficient to evaluate long-term graft incorporation and durability. The small sample size (n=14) limited the ability to perform subgroup comparisons. Another limitation was the lack of objective knee laxity measurement using a laxometer or radiological laximetry; assessment was limited to clinical grading. Vibration testing was not feasible as preoperative data were unavailable. Data on the time taken to achieve normal gait, podogram analysis, and muscle power recovery could not be collected because follow-up was performed only once. Standard knee scoring systems such as International Knee Documentation Committee (IKDC) were not used. Repeat arthroscopy to assess graft incorporation or remodeling was not performed. The cause of graft necrosis in two patients was unclear.

In the statistical analysis of the one-leg hop test, the observed mean difference (−1.92 inches) was almost twice the initially assumed difference (1 inch) used for sample size calculation, indicating that the study was underpowered for the actual effect size. Although the results remained statistically significant, this deviation from the original assumptions warrants caution and underscores the need for larger studies with more precise power estimations and correlation with validated knee function scores to confirm these findings.

## Conclusions

Our findings suggest that fresh frozen soft tissue allografts appear to be a viable alternative to autografts for ACLR, particularly in the Indian population, where most ACL injuries occur in non-athletic individuals. In our cohort, there were no cases of disease transmission or graft re-rupture, and most patients achieved satisfactory functional outcomes. However, rare graft necrosis highlights the need for close postoperative monitoring. The observed mean difference in one-leg hop performance was nearly double the initially assumed value used for sample size calculation, indicating that the study was underpowered for the actual effect size, although results remained statistically significant for non-inferiority. These findings, while encouraging, should be interpreted with caution, and larger, adequately powered studies are needed to confirm outcomes, especially in athletic populations. The expansion of tissue banks and the development of standardized national guidelines will be essential for the safe and widespread adoption of allografts in orthopedics and sports medicine.

## Appendices

**Table 4:** Proforma RTA: Road Traffic Accident; ROM: Range of motion; HA: Hydroxyapatite; TCP: Tricalcium phosphate; ACLR: Anterior cruciate ligament reconstruction. (The proforma was created by the authors incorporating concepts from established knee outcome measures [12-15])

Patient details	
Name	Mr -
Age/Sex	-/-
Hospital inpatient number	-
Address	-
Phone number	-
Injury details	
Chief complaints	Pain and instability/ Locking
Mode of injury	Sports related/ RTA/ Slip and fall
Time since injury (in months)	-
Side affected	Left/ Right
Preoperative examination details	
Knee ROM	-
Quadriceps power	-/5
Lachman test	-
Anterior drawer test	-
One-leg hop test	-
Perioperative details	
Date of surgery	-
Allograft tendon used	Peroneus longus/ Peroneus brevis/ Tendoachilles/ Semitendinosus/ Others............
Technique used	Single bundle/ Double bundle/ Triple weave
Fixation method used	Titanium screws/ HA coated TCP screws
Postoperative remarks	
Questionnaire related to complaints	
Do you have pain in the operated knee?	If yes, kindly mention onset of pain, how frequent is the pain, role of rest in pain management and encircle the severity of pain in the VAS scale.
Do you have swelling in the operated knee?	If yes, kindly mention the activity leading to swelling and the frequency of such episodes.
Do you have instability in the operated knee?	If yes, kindly mention the specific activity leading to instability and the frequency of such episodes.
Do you have locking in the operated knee?	If yes, kindly mention the specific activity leading to locking and the frequency of such episodes.
Do you have any weakness in the operated limb?	If yes, kindly mention which specific activity reveals weakness in the operated limb.
Do you limp with the operated leg?	If yes, kindly mention whether the limp is associated with pain or not.
Questionnaire related to function	
Are you able to walk normally?	Yes/ No
Is it possible for you to jog or run up to one km without difficulty in the operated leg?	Yes/ No
Are you able to climb upstairs without support?	Yes/ No
Are you able to climb downstairs without support?	Yes/ No
Are you able to drive two-wheeler without difficulty?	Yes/ No
Are you able to use public transport without difficulty?	Yes/ No
Are you able to sit crossed leg without difficulty?	Yes/ No
Are you able to squat without difficulty?	Yes/ No
Questionnaire related to satisfaction from Allograft ACLR	
How do you rate your satisfaction from the surgery?	Excellent (or) Good (or) Fair (or) Poor
Will you recommend allograft ACLR for people known to you with similar problem?	Yes/ No
Will you undergo allograft ACLR if you happen to get similar problem again?	Yes/ No
Do you participate in sports activities?	If yes, kindly mention the sport and frequency of involvement in sports.
Clinical examination at follow-up	
Inspection	-
Palpation	-
Movements	-
Measurements	-
Muscle power: Quadriceps and Hamstring	-/5
Lachman test	-
Anterior drawer test	-
Posterior drawer test	-
Varus/valgus stress test	-
Pivot shift test	-
Tests for meniscus	-
One-leg hop test	Left - and Right -
